# “The System Tends to Scoop You Up and Spit You Out and They’re Done With You”: The Intersection of Intellectual/Developmental Disability and Homelessness From the Perspectives of Service Providers

**DOI:** 10.1177/10497323231186880

**Published:** 2023-08-07

**Authors:** Whitney Thurman, Elizabeth Heitkemper, Tara Hutson, Angela Preston, Jonathan Hecht

**Affiliations:** 1 School of Nursing, 12330 The University of Texas at Austin, Austin, TX, USA

**Keywords:** disability, homelessness, systems coordination, system churn

## Abstract

People with intellectual and developmental disabilities (IDD) experience elevated risk for poor health and social outcomes in adulthood and are at risk for experiencing homelessness and housing instability. Although the exact prevalence of IDD among homeless populations is unknown, a small body of literature related to the intersection of IDD and homelessness suggests differential health needs and service use patterns, with a need for targeted health and social services. In this study, we explore the perceptions and experiences of 18 homeless or disability service providers about (a) their clients at the intersection of IDD and homelessness and (b) their role and the services provided at the intersection of IDD and homelessness. Participants struggled to provide appropriate, accessible services for this population, owing to lack of training and awareness of specific needs, fragmented systems, and inadequately funded healthcare and housing support. Our findings also reveal that clients at this intersection have high contact with public systems, which places them at risk for losing their right to self-determination. Recommendations center on systems transformation to facilitate the ability of providers to collaborate and to make data-driven decisions to deliver person-centered care.

## Introduction

Every year, 2.5 to 3.5 million Americans sleep in shelters, transitional housing, and public places not meant for human habitation; in other words, they experience homelessness (Henry et al., 2022). Almost 50% of people experiencing homelessness (PEH) live with some type of disability, at a rate 2.5 times higher than that of the general U.S. population (19.8%; Henry et al., 2022). Of particular concern are the PEH with intellectual and developmental disability (IDD).

Intellectual and developmental disability is characterized by impairment of cognitive functions in at least three of the following areas: language, learning, mobility, self-direction, capacity for independent living, economic self-sufficiency, and self-care (Carulla et al., 2011). Common conditions that fall under the IDD umbrella include cerebral palsy, autism spectrum disorder, learning disability, and Down syndrome. These IDDs last throughout a person’s life and occur across all racial, ethnic, and socioeconomic groups, and the number of individuals aging with these lifelong IDDs is expected to double by 2030 (Factor et al., 2012).

Despite the many strengths of people with IDD (PWIDD) and the fact that many live healthy, meaningful lives, PWIDD experience heightened risk for poor health outcomes in adulthood. For example, PWIDD are at increased risk for morbidity and premature mortality due to complex physical and mental health conditions (Landes et al., 2019; Schoufour et al., 2018). Poor mental health is common for PWIDD and is frequently attributable to early adverse life events such as trauma and abuse (Koslowski et al., 2016). Among adults with IDD, poor mental health is also associated with communication differences that complicate the ability to obtain a diagnosis and appropriate care (Whittle et al., 2018). Diagnostic overshadowing, whereby changes in an individual’s behavior are attributed to a person’s IDD without consideration of underlying physical or mental health conditions, is another important contributor to missed diagnoses and inappropriate treatment plans in this population (Baxter et al., 2006; Mason & Scior, 2004). It is also important to note that PWIDD have been actively excluded from much of the research that underpins medical and public health practice (Brooker et al., 2015), suggesting that even with appropriate access to medical care, the needs of this population may not be met.

Under current systems of care, PWIDD also experience disproportionately poor social outcomes in adulthood (Howlin et al., 2013; Howlin & Moss, 2012). In the United States, for example, only 14.7% of PWIDD are employed, as opposed to 71.4% of adults without disabilities (National Core Indicators, 2018). Accordingly, many PWIDD live in poverty and rely on publicly funded income such as Supplemental Security Income (SSI) and housing support to meet living expenses. SSI pays an average of $841 per month, and there is no housing market in the United States where such an income can provide a safe, decent rental unit (Schaak et al., 2017). This confluence of factors creates substantial risk for PWIDD to experience homelessness.

The exact prevalence of IDD within the population of PEH is unclear, but estimates range from 12% to 39% (Durbin et al., 2018). The pathways into homelessness for PWIDD are complex, and as with homelessness more broadly, risk factors arise from an array of individual, social, economic, and political influences. Common factors that contribute to homelessness among PWIDD are low educational attainment, challenging behaviors, and the breakdown of family relationships (Mercier & Picard, 2011; Morton & Cunningham-Williams, 2009; Oakes & Davies, 2008; Van Straaten et al., 2014). Other issues that have been identified as contributing to homelessness in this population include the caregiving burden and stigma associated with having a family member with IDD, leading to instability in the family home (Mitter et al., 2019).

The service use and care needs of PEH are well documented (Madigan & Friedman, 2021; Subedi et al., 2022), as are their high rates of mental illness (Fazel et al., 2008), problematic substance use (Fazel et al., 2008), and physical health problems (Havercamp & Scott, 2015). In the United States, the homelessness response system (HRS) in a given community is composed of non-profit organizations, housing authorities, public health departments, and other governmental entities each of whom has responsibility for providing specific services. An individual experiencing homelessness must navigate this system to find the distinct organizations that provide for needs such as food, water, and shelter. System navigation is done with the assistance of a caseworker who can assess their eligibility for housing resources and help to obtain other services such as healthcare or employment support. However, given the limited resources and high burden of poor health among PEH, navigating this sprawling and fragmented system poses substantial challenges (Thurman et al., 2022). The small body of literature related to IDD and homelessness suggests that individuals in this intersection experience more psychological distress and substance use than do those without IDD (Van Straaten et al., 2014), posing the possibility that this subgroup of PEH may have added difficulty navigating the HRS and suggesting a need for targeted health and social services for this subgroup of PEH. It remains unclear how well the HRS and services for PEH are able to address the needs of PWIDD who are experiencing homelessness.

### Purpose

Given the complex confluence of factors facing PWIDD who also experience homelessness, it is important to understand the extent to which homeless service and disability service providers recognize unique needs and are equipped to care for and provide services to this population. The purpose of the present study, therefore, is to examine the perceptions and experiences of service providers about (a) their clients at the intersection of IDD and homelessness and (b) their role and the services provided at the intersection of IDD and homelessness. Service providers working with PWIDD will provide a critical insight into the issues currently faced by PWIDD who experience homelessness and the systems through which they must navigate. Findings from this study will provide a solid foundation for future work to engage PWIDD to further explore this area.

## Method

Because little was known about service providers’ knowledge, awareness, and perceptions of the intersection of IDD and homelessness, for this study we used a qualitative descriptive design with inductive content analysis (Hsieh & Shannon, 2005). This approach is well-suited to exploratory research (Schreier, 2012) because it elicits detailed description and descriptive validity through the examination of text that represents individuals’ experiences and perspectives (Sandelowski, 2010). The study was deemed exempt by our university’s Institutional Review Board (Study 00000755). Participants provided written informed consent prior to study enrollment.

### Sample, Setting, and Recruitment

Providers were eligible for participation if they were aged 18 years or above and had been involved in the provision of homeless services or disability services for at least 12 months. Purposive sampling was used to identify individuals who had been providing services to PEH, people living with IDD, or both, for at least 1 year. If, upon contact from the research team, a potential participant agreed to learn more about the study, the team sent an informational email providing the study’s purpose, with details and expectations. The email included a link to a Qualtrics survey that contained relevant information and an option to assent to participate. Upon the participant’s agreement, the survey proceeded with demographic questions and scheduling of interviews. For those who did not provide assent, the survey ended by thanking them for their consideration and time. Interviews occurred between March and June 2021. Participants received a $40 gift card in appreciation of their time and expertise.

### Data Collection

Interviews were conducted remotely using Zoom. The first author of this study independently conducted 10 interviews, the second author independently conducted seven, and the two of them conducted one interview together. Each interview lasted approximately 1 hour. The interviews were recorded, and the audio recordings were professionally transcribed. Data saturation was approached with interview 13. Interviewers met to discuss any new findings after each subsequent interview and agreed that data saturation was ultimately reached.

### Data Analysis

Inductive qualitative content analysis (Schrier, 2012) provided detailed descriptions of participants’ knowledge, awareness, and perceptions of the intersection of IDD and homelessness. Data analysis was facilitated with the use of the qualitative data management program NVivo. To begin, the coding team as a group coded one interview, discussing meanings and describing the data with initial codes. Next, the five researchers independently coded a second transcript using the initial codes and adding new ones as needed. The coding team then reconvened to compare coding, to review and discuss the meanings and uniqueness of the initial codes, and to develop a coding scheme. The coding scheme then formed the basis for subsequent coding in which pairs of researchers independently coded two transcripts before meeting as pairs for review. After two rounds of this process, the large group reconvened for review to reach consensus. At that point, the first three authors divided the remaining 12 transcripts and coded them independently. Upon completion of coding, the research team met to ensure that all codes approached meaning saturation (Hennink et al., 2017).

### Rigor, Validity, and Reliability

Procedures for credibility, transferability, dependability, and confirmability were incorporated throughout this research to ensure trustworthiness. These procedures included taking field notes, team debriefing, reflexive journaling, consideration of negative cases, and maintenance of an audit trail using NVivo.

## Results

### Sample Demographics

Table 1 presents the demographic characteristics of the participants (*N* = 18). The sample represented both homeless and disability service providers.

**Table 1.** General Characteristics of Participants (*N* = 18).

Characteristics	
Gender, *n* (%)	
Female	11 (61.1)
Male	7 (38.9)
Type of practice, *n* (%)	
Homeless services	9 (50)
Disability services	8 (44.4)
Both	1 (5.6)
Profession, *n* (%)	
Case manager	7 (38.9)
Outreach specialist	1 (5.6)
Allied professional	5 (27.7)
Attorney	2 (11.1)
Administrative	2 (11.1)
Behaviorist	1 (5.6)
Clinician	5 (27.7)
Psychiatric mental health nurse practitioner	2 (11.1)
Registered nurse	1 (5.6)
Physician	1 (5.6)
Licensed psychologist	1 (5.6)
Age in years, *M* (SD)	44 (13.9)
Time with employer in years, *M* (SD)	5.8 (6.3)
Homeless services	3.2 (2.1)
Disability services	8.8 (7.9)
Both	11

## Qualitative Findings

### Participants’ Perceptions of Clients

Four themes revealed perceptions and experiences of participants about their clients at the intersection of IDD and homelessness: *difficulty with daily and long-term management, socialization is challenging, moments of extreme vulnerability, and high risk for trauma and abuse* Disability service providers typically discussed individuals with a confirmed IDD diagnosis who were precariously housed and who cycled in and out of homelessness. In contrast, homeless service providers described experiences with clients who were experiencing homelessness but often lacked a formal IDD diagnosis despite strong clinical suspicion of such. Both groups were able to speak to numerous experiences with clients both known and suspected to be at this intersection.

#### Difficulty With Daily and Long-Term Management

This theme encompassed difficulties that complicated efforts of participants and their clients to manage daily life and to plan for the longer term; categories included *medications and health needs*, *unknown histories*, *challenging behaviors*, and *accessibility barriers.*

##### Medications and Health Needs

Participants recognized that the complex health challenges that sometimes accompany IDD and that are associated with homelessness were exacerbated for their clients at this intersection. Difficulty managing medications was a specific issue mentioned due to both physical and cognitive limitations of IDD as well as the structural barriers posed by homelessness. Because of these barriers to optimal medication adherence, participants said that chronic health conditions were poorly managed: “the physical needs differ because their diabetes and high blood pressure are—tend to be out of control, because they’re not taking their meds*.*” Participants said that this contributed to further disability and additional difficulties with completing activities necessary to manage health, meet social needs, and exit homelessness.

##### Unknown Histories

Participants struggled to gain a complete understanding of their clients’ circumstances and needs, thereby hampering their ability to provide appropriate medical and social services. As one participant said, “a lot of the time…we’re trying to determine, like, was this a TBI—a traumatic brain injury—or is this IDD because it’s really hard to differentiate sometimes if you don’t have those early on school records.” Efforts were complicated by fragmented or unavailable medical and educational records, combined with incomplete histories obtained from clients. One participant described working with a client to gather his school records. She said, “they shared with me the names of their schools…none of those schools existed. And I am still having difficulty tracking [them] down.”

##### Challenging Behaviors

Several participants described challenging behaviors that created conflict between clients and their environments. Participants described aggressive, self-injurious behaviors and typically recognized that they stemmed from the mismatch between clients’ disabilities and complex medical issues and the social environments in which they lived. Participants believed that their clients with IDD were generally misunderstood, creating communication barriers that often manifested as socially unacceptable and potentially dangerous behaviors. One participant shared her perception that “people can sometimes perceive them [people with IDD] as rude or verbally aggressive-type people.” Subsequently, much time and effort were spent on trying to manage these behaviors.

##### Accessibility Barriers

Every participant described some type of challenge that their clients encountered when attempting to access needed services and support. Many of these barriers were related to activities critical to daily life. For example, transportation posed unique challenges; participants shared that many of their clients were unable to navigate public transit independently. Participants also described access barriers stemming from inadequate communication technologies. It was commonly reported that many clients struggled with smartphone technology, owing either to not having a working phone or to being unable to use it effectively. One participant described coaching a client to answer his smartphone as follows: “he knows how to make calls, but no matter how many times we’ve gone over how to answer the phone, he’s not grasping that.”

Other barriers were caused by the severely under-resourced mental health system in the state of Texas and the dearth of available resources to support adults with IDD to live independently in the community. Lack of health insurance was a specific access barrier that undermined clients’ ability to obtain needed healthcare services and that limited available housing options.

#### Socialization Is Challenging

The second theme involved the constellation of challenges experienced by clients as they attempted to navigate normative systems. Participants from both homeless and disability services recounted coaching clients regarding societal expectations. They also shared consequences of their clients’ failing to adhere to these expectations, which ranged from losing employment to remaining institutionalized to being kicked out of their homes. The categories within this theme were *combination of high-acuity health and social needs*, *difficulty with traditional social norms*, and *developing community within homelessness.*

##### Combination of High-Acuity Health and Social Needs

Every participant described a constellation of complex, high-acuity health and social needs that typified their patients and clients at the intersection of IDD and homelessness. These included chronic health conditions, serious mental illness, substance use, and trauma-related mental health struggles. One participant described her client population as follows: “schizophrenia, schizoaffective, you know, all these big disorders. And I’m like, okay, you know, it’s teasing out that aspect because there are parts of IDD that can look behavioral or can look psychiatric but they’re not, you know?” When asked about his perception of the prevalence of substance use in this population, one participant said, “oh, omnipresent…you know, the exception is when it’s not present…folks are trying to get by and medicate themselves and do what they can.” Participants also perceived that these high-acuity needs often precluded their clients’ ability to engage in activities of daily living such as basic hygiene, contributing to stigmatization and further difficulties with socialization.

##### Difficulty With Traditional Social Norms

Participants described ways in which their clients would not or could not adhere to traditional social norms and were subsequently marginalized or, frequently, institutionalized. For example, participants referred to clients’ preferences for unorthodox hobbies and atypical daily schedules, which caused conflicts with peers, neighbors, and family members. One participant put it this way: “my perception of it is more, in regards to people…getting into trouble because they’re not able to necessarily understand consequences.”

Overall, participants recognized and lamented the ways in which growing up with IDD in American society creates enormous challenges for healthy growth and development and socialization into behavioral norms for adults. One clinician who worked primarily with adults with IDD elaborated on this: “people with IDD have often been on the fringes of society. If they lived at home and grew up with their parents, they may have been protected, but in the school setting they may have been ostracized, bullied, certainly left out…there’s all kinds of language in those systems that demeans that person.”

##### Developing Community Within Homelessness

Participants perceived that for a certain subset of individuals at this intersection, being a member of the homeless community provided a sense of belonging that was difficult for them to find otherwise. As one clinician said, “oftentimes when they go to the streets, they’re just another person…that culture system is open in a different way for different abilities and eccentricities.” Others described similar experiences with clients who struggled to adapt to group home settings and cycled in and out of homelessness.

#### Moments of Extreme Vulnerability

Moments of extreme vulnerability encompassed periods of time when clients—vulnerable at baseline—experienced heightened vulnerability due to external forces. Both homeless and disability service providers described ways in which shifts in the life of a client could have devastating ramifications many years hence. Sometimes these shifts were unanticipated, but often they were expected outcomes of unfolding processes for which clients had not been adequately prepared. The categories within this theme include *changes in caregiver capacity* and *transitions through fragmented systems*.

##### Changes in Caregiver Capacity

Participants recounted several instances of clients with IDD losing their housing when their parents or caregivers were no longer able to care for them. Often this was due to the death of a parent. One participant described her client’s progression into homelessness in the client’s own words: “Well, after my mom died, you know, I was living in the house for a year by myself and then the house got condemned. And, and now I’m homeless.”

##### Transitions Through Fragmented Systems

Participants lamented the fragmented systems within which they worked and shared that their clients existed in a nearly perpetual cycle through these systems. With every institutionalization and subsequent discharge, clients were at heightened risk for losing contact with service providers, family, and friends, contributing to an array of poor outcomes that left participants scrambling to piece together an understanding of discharge instructions and treatment plans. One healthcare provider declared that it took “*an act of Congress*” to get any information from inpatient hospitalizations for her patients. The criminal legal system was another system mentioned specifically, with one participant explaining that there was little to no community discharge planning: “the system tends to scoop you up and spit you out and they’re done with you.”

#### High Risk for Trauma and Abuse

All participants recognized that individuals at the intersection of IDD and homelessness often rely on the support and goodwill of others to meet daily needs, safeguard possessions, and navigate complex systems. They connected this precarious position of being dependent on others to heightened risk for trauma and abuse, and they shared troubling stories from their clients’ lives. Categories within this theme include *at risk for exploitation*, *vulnerable due to homelessness-related factors*, and *guardianship—a double-edged sword*.

##### At Risk for Exploitation

Participants described ways in which individuals with IDD were at risk for exploitation regardless of housing status. Disability service participants explained that many of their clients with IDD required support to manage their money and to make informed medical decisions. Both disability and homeless service participants perceived that patients with IDD could not effectively self-advocate for needed services or resources. This combination of factors places individuals at this intersection at high risk for being exploited by ill-intentioned others—family, friends, and strangers—who took advantage of them in myriad ways. One case manager spoke of a particular client with whom she had worked: “I have no idea how he was surviving outside. He is extremely cognitively impaired and extremely vulnerable. Just will do whatever somebody asks him to do.”

##### Vulnerable Due to Homelessness-Related Factors

Homeless service providers described how the precariousness of homelessness increased vulnerability among individuals with IDD. Being victimized is a common experience among unsheltered individuals, and participants routinely described their clients’ possessions being stolen. Participants from both groups shared distressing accounts of clients being trafficked. One participant said, “I would say the people I can think of who I know are IDD on the streets…were [more] vulnerable to like substance use. And then, also getting exploited on the street or getting taken advantage of.”

##### Guardianship: A Double-Edged Sword

Guardianship was discussed by the majority of participants. Participants acknowledged that some of their clients with IDD benefited from having a guardian to help them with decision-making: “I had this one patient…she should’ve never been released from the state hospital. She is a danger…We, actually, the dad had to get guardianship, move her out of the state…to get her the help she needed.” However, they also described difficulty due to lack of family support: “we’ve tried to encourage family [to] act as guardians…you know, but that is very difficult because that’s part of what contributed to why they’re in this experience anyway is that their family ties are virtually non-existent.” Participants also recognized the tensions inherent in respecting clients’ rights to self-determination and guardians’ efforts to mandate that their charges live in group homes or other institutional settings.

### Participants’ Perceptions of Roles and Services

Five themes revealed providers’ perceptions of their own roles in working with individuals at the intersection of IDD and homelessness: *provider’s role consumed by detective work, disability benefits—the main goal, services are limited, help wanted,* and *metric misalignment impacts providers.*

#### Provider’s Role Consumed by Detective Work

The first theme for providers’ perceptions of their own roles encompassed their efforts to piece together medical and social histories in order to understand and best meet clients’ needs. Categories within this theme include *identifying causes of cognitive impairment*, *seeking historical records*, and *trial and error medication management.*

##### Identifying Causes of Cognitive Impairment

Because a substantial percentage of the individuals whom the providers served experienced some type of cognitive impairment but lacked a diagnosis, the participants who worked in homeless services described the protracted process of identifying underlying causes of their patients’ cognitive impairments. Clinicians endeavored to identify biological processes or acquired injuries that might be impacting cognition by ordering lab tests, neurological screening, and imaging studies. One clinician described working with a particular patient who had been referred to her by case management:

we just didn’t have a lot of information on him. And, so, they brought him to me saying, “Can you, you know, do any tests or figure out where—you know, why he’s cognitively impaired? Is there a traumatic brain injury? Did he have a stroke? Or is this IDD? Like, you know, do we know?” Um, so, we basically just had to, like, kind of be a detective.

##### Seeking Historical Records

Because IDD services require a diagnosis of IDD before the age of 22, participants working in homeless services spent much time trying to obtain historical records. Most often, they described processes for seeking school records that might confirm a diagnosis of IDD or receipt of special education services while in primary or secondary school. One homeless service case manager described working with one client:

a gentleman in his 50s, who we believe has IDD…has no ability to recall information. Um, we actually had to figure out his last name because when I first met him, he reported two different last names that were similar, but different, and three dates of birth. So, it actually took six months just to figure out this person’s identity.

##### Trial and Error Medication Management

The comorbid health conditions that individuals at the intersection of IDD and homelessness often have necessitate complex medication regimens. However, because of clients’ instability and unpredictability, both the homeless and the disability service participants explained their careful approach to prescribing medications and monitoring adherence. One clinician put it thus:

I do say that they’re just more unpredictable and a lot harder to treat ’cause you also have to take safety into account too. I can’t just be medicating a patient that, one, is not gonna show up for medication. And now I’m gonna put them in withdrawal. Or, um, the type of medication they’re taking is not safe to go out.

#### Disability Benefits: The Main Goal

The next theme for service providers’ perceptions of their roles was related to disability benefits. This theme permeated homeless service providers’ responses, in that the necessity of disability benefits and the effort to obtain them colored their perceptions of their clients as well as of their own professional roles. Because most of the disability service providers with whom we spoke cared for individuals with an IDD diagnosis who were already receiving some type of disability benefits, they recounted the importance of those benefits, but their own roles were not dominated by the pursuit of such. Within this theme, categories included *difficult process to navigate* and *critical first step*.

##### Difficult Process to Navigate

Participants described what they believed to be the tedious work of applying for, and subsequently managing, disability income and associated medical benefits for their clients. Benefits applications were time consuming, and the process was made substantially more difficult by a lack of needed documentation and the absence of family who could fill in gaps in knowledge. Participants uniformly lamented the byzantine procedures involved with this process. One participant put it succinctly: “To get an IDD appointment through [the local mental health authority] is the biggest nightmare I’ve ever seen.” A disability service case manager shared her process of documentation thus: “this determines their funding. So, I don’t want to put something that’s going to get, like, money taken away from them. I want to be able to give them as much assistance as they need.”

##### Critical First Step

Participants clearly understood that despite the arduous application process, disability benefits were the clearest and quickest route to opening avenues for housing support and healthcare services that would otherwise remain unattainable. They also believed disability benefits to be the only route to income for this population of clients. In describing his experience with one client, a case manager recounted, “Getting a job has really proved to be not an option…the only reasonable income plan for them is to get disability…because we can house people without income, but if they don’t have an income plan, like a feasible income plan, there’s really no sense in housing them.”

#### Services Are Limited

Extremely limited services were available for individuals at this intersection. Categories included *known barriers: cross-system coordination and collaboration* and *no targeted services.*

##### Known Barriers: Cross-System Coordination and Collaboration

Participants expressed frustration with the lack of interdisciplinary collaboration and described archaic processes for obtaining and sharing records. These processes frequently included faxing hard copy release forms and waiting for records to be sent via U.S. mail in order to gain access to needed information. After describing how she incorporated these processes into her daily work, one homeless service provider chortled and said *“no”* when asked whether there were any local organizations that did this well. Another participant described the regular occurrence of inpatient psychiatric facilities being completely unaware of an individual’s admission to a different facility within the community—even of instances that had occurred just days or weeks prior.

##### No Targeted Services

Participants denied any knowledge of services that were targeted for people with IDD who were experiencing or were at risk for experiencing homelessness. One case manager from a safety-net healthcare clinic said, “we don’t have any IDD services. It’s not talked about in our agency; it’s not available.” A disability service case manager shared a slightly different perspective. She spoke of a client who had been hospitalized with COVID but was receiving substandard care due to the hospital staff’s lacking knowledge of best practices to care for individuals with IDD: “for individuals I serve, their structure, their day-to-day life, it needs to be exact. And if it’s not exact, they are going to experience challenges…they don’t know how to care for individuals like ours.”

#### Help Wanted

No participants reported having received specific training for working with individuals with IDD; their desires were summarized by the categories of *formal training wanted* and *desire for new tools and interventions.*

##### Formal Training Wanted

Trial and error constituted participants’ typical approach to identifying available resources and understanding the needs of their clients. Many expressed frustrations with this tactic. As one case manager said, “you know, if you don’t have experience with it, you’re just kind of guessing. And I don’t want—I don’t like to guess.” Participants identified communication techniques, trauma-informed care, understanding clients’ social needs, and identifying and accessing available resources in which formal training would have improved their ability to provide care and support for PWIDD who were experiencing homelessness or housing instability. One participant distinguished between what had been available to her as a home healthcare nurse with what is available to her in her present position working with adults with IDD:

I think it’s very important that we get, um, enough training so that we can really support these people. Uh, because there’s not really, like, a good training out there. For example, I came from Home Health, you know. There were lots of trainings back and forth, back and forth. Well, here, there’s not a lot of trainings. There are no even um, support, like…all these things that I could go to to help myself.

Participants also identified others along the continuum of disability and homeless services—including law enforcement officers and judges—who needed training specific to this population.

##### Desire for New Tools and Interventions

Participants believed that innovative solutions could make their jobs easier, that remote access to client’s records could facilitate case management and provision of other services, and that more robust mobile technology solutions would be helpful as well. The latter included online portals through which providers could remotely access case management notes and health records, a mechanism that would grant permission to obtain information in real time, and technology-based checklists that could be used to help manage activities of daily living.

#### Issues With Data and Outcomes

This final theme for participants’ perceptions of roles and services included the following categories: *data problems result in minimal data-driven insights* and *predefined metrics.*

##### Data Problems Result in Minimal Data-Driven Insights

Participants described their data systems and documentation practices, with disability service providers reporting use of various systems including electronic health records, paper charts, and personally developed databases. Most of the homeless service providers reported using their organization’s documentation system and the Homeless Management Information System (HMIS). Both groups described issues with their data systems and lamented the resulting poor data quality: “Our data is only as good as the people who enter it, so our data quality isn’t the greatest.”

Specific to participants working in homelessness were issues with the federally mandated HMIS, which many homeless service providers who work in the same local community can access. Participants described how HMIS was rarely used to collect robust individual-level data, and few providers reported accessing clients’ records during face-to-face encounters. “I’m not sure how it works…we really don’t upload anything into it.” This prevented participants from using data to inform client care in a holistic way and contributed to the sentiment that HMIS was not useful in practice. However, participants did recognize that with better implementation, HMIS could be a useful tool: “it would be nice if we could upload maybe medical information about a client just because that is…the biggest barrier to housing is either their medical issues or lack of documentation of their medical issues. It would be nice if that could be more of a shared resource.”

##### Predefined Metrics

Participants shared frustrations regarding metrics that their own agencies employed as well as the metrics used by agencies with whom they partnered. Participants perceived that benchmarks were predetermined by agency leadership in response to funding requirements. One participant thus stated that “the caseworkers get less say and then it becomes all about like billing.” Participants also believed that a lack of qualitative data to document clients’ perspectives as well as their own experiences was troublesome and that predefined metrics impacted the services they could provide and the agencies to which they could refer clients. Subsequently, it was difficult for participants to perceive much benefit from the often tedious but mandated reporting requirements.

## Discussion

Little evidence regarding the intersection of homelessness and IDD in the United States exists, and the results of this study extend our understanding of this population in several ways. First, health and social service providers perceive a substantial prevalence of IDD among the population of people experiencing homelessness as well as a considerable amount of housing instability among PWIDD. These findings support prior evidence documenting a disproportionate overrepresentation of IDD among PEH (McKenzie et al., 2019). Our findings also indicate that while health and social services recognize that this population has unique needs, providers do not feel equipped to meet those needs. Further, providers perceive that the systems within which they work are not structured in a way that would allow this population to easily have their needs met even if providers themselves were better equipped. These findings suggest the utility of the twin-track approach to systems design endorsed by the United Nations Convention on the Rights of Persons with Disabilities (UNCRPD) (United Nations, 2009). The twin-track approach promotes accessibility and inclusion throughout public systems while also ensuring that the professionals who work within those systems are fully equipped to meet disability-specific needs.

Our findings reveal that although many of the challenges facing PWIDD who are experiencing homelessness are similar to those for other PEH, this subpopulation also has distinct needs. In particular, the lack of accessibility within public systems such as hospitals, emergency services, social security, and the criminal legal system, combined with a general lack of awareness among professionals about the unique needs of PWIDD, contributes to a heightened risk for unmet health and social needs in this subpopulation. The COVID-19 pandemic exacerbated many of these risks and compounded the difficulty experienced by participants when trying to support clients to navigate through the complex HRS. The current study did not specifically examine the impact of COVID-19, but future research could investigate the influence of the pandemic response on these systems and on the ability of PWIDD to access them.

Individuals at the intersection of IDD and homelessness exist in a nearly perpetual process of cycling through systems, a phenomenon known as system cycling or system churn (Harding & Roman, 2017). The mismatch between individual needs and the structure and funding of human services results in individuals with impaired cognitive capacity having frequent contact with these systems (Clapton et al., 2014). Early evidence from Canada has identified key ingredients of a cross-sector partnership between housing, health, and intellectual disability sector organizations that was designed to support PWIDD who are experiencing homelessness (Lamanna et al., 2020). Critically, their first step was to streamline access to neuropsychological assessments of homeless shelter users who were suspected of having an intellectual disability in order to connect individuals with appropriate resources. Lessons learned from the project included early struggles with interdisciplinary communication and collaboration. Future research could refine this partnership model and measure its success in supporting PWIDD to remain housed and in reducing system churn. Future policy efforts should prioritize the reduction of system churn by funding efforts that replace fragmented systems with a cohesive health ecosystem.

There is scant evidence for how to best support PWIDD as they navigate the complex landscape of siloed systems after reaching adulthood. This lack of understanding contributes to a continued reliance on guardianship, whereby individuals who are judged to be incapacitated or impaired are stripped of their legal rights and assigned another person to make all decisions on their behalf. Guardianship is increasingly recognized as stigmatizing and contradictory to the human dignity of those who are subjected to it (Gooding, 2013) because it disregards the ethics of promoting autonomy and the right to self-determination. Recent estimates have put the rate of guardianship in adults with IDD in the United States as high as 89% (Bradley et al., 2019).

Supported decision-making (SDM) has emerged as a viable alternative to guardianship, and evidence suggests its utility in ensuring that the individual’s preferences are taken into consideration and respected as much as possible (Blanck & Martinis, 2015; Jameson et al., 2015). SDM is a process with which a third party helps or supports individuals with intellectual or cognitive disabilities in making legally enforceable decisions for themselves instead of removing their right to make their own decisions (Kohn & Blumenthal, 2014). There are various SDM models, but all of them rely on an individualized process whereby people with disabilities are supported by friends, family members, or professionals to give them support to understand their own needs and make their own decisions (Blanck & Martinis, 2015). Regardless of the particular SDM model used, the individual with a disability has a trusted individual on whom to rely. Future research should explore how to translate SDM for populations that lack robust support networks, such as those who experience homelessness and housing instability. Research could also explore the impact on health and housing outcomes if SDM is implemented earlier during the life course while PWIDD are stably housed or have closer family connections. Finally, future research should also examine how to better integrate aspects of support and autonomy into our systems such that all people are able to get the comprehensive care they deserve.

Service providers in the present study overwhelmingly described how a bulk of their time was spent in contacting providers in other systems to obtain historical records, and they highlighted barriers that they faced when they engaged in this process, which were due in part to difficulties with data sharing. Antiquated documentation systems lacked interoperability and hindered interdisciplinary collaboration, holistic understanding of clients, and organizational understanding and decision-making. Our findings, therefore, support and extend earlier calls for data systems to be integrated among housing, disability, and healthcare sectors (ECONorthwest, 2020). Linking data from state and local agencies that interact with and serve individuals with IDD, including state disability agencies, education departments, and those entities responsible for Medicaid and other public benefits, would not only improve our understanding of the health and service needs of the population but also greatly improve individuals’ ability to access and receive needed services and support. In the United States, the Health Insurance Portability and Accountability Act (HIPAA) protects sensitive patient health information from being disclosed without consent, and the UNCRPD provides a comprehensive framework for data protection and ensuring the right to privacy that extends beyond protected health information (United Nations, 2009). Because persons with disabilities are at high risk of having their privacy breached and of experiencing discrimination based on their impairments, it is critical that all efforts to improve data infrastructure are made in accordance with this human rights–based approach to data collection, privacy, and access to information. Future policy efforts need to fund this crucial effort to create a unified health ecosystem that is not hampered by data and communication issues.

Participants spoke of the sense of belonging and community that people reported developing while they were homeless but struggled to honor and replicate in the housing alternatives typically available to them. Gaboardi et al. (2021) have examined community integration from the perspective of PEH and found that integration consists of more than a particular set of behaviors; it also has emotional components that are rarely addressed. Gaboardi et al. argue that personal dignity, respect, and recognition from others are critically important for PEH’s ability and desire to integrate into their larger communities. For PWIDD experiencing homelessness, it is likely that these emotional needs are even more critical. It is well documented that feelings of loneliness are common among PWIDD (Alexandra et al., 2018) and that stigma is a major barrier to acceptance and inclusion regardless of culture (Jansen-van Vuuren & Aldersey, 2020). Future work should examine the perspectives of PWIDD who are experiencing homelessness so that services can be better designed to foster community and belonging. Furthermore, this work should be used to facilitate changes in policies and social norms to help society see the value inherent in disabled individuals.

### Limitations

In this study, we investigated the experiences and perceptions of a group of homeless and disability service providers with regard to their clients at the intersection of IDD and homelessness in Texas. The study’s small sample and its setting reflect conditions specific to the participants and are not generalizable to all homeless and disability service providers. However, we have addressed transferability by providing detailed descriptions of the participants’ data and context and by using direct quotations. Additional strategies to enhance trustworthiness included validating interpretive claims during regular team meetings. It can be seen as a limitation that the researchers did not verify interpretations with the study participants, and it should also be noted that no individuals with IDD participated in the study. Understanding their perceptions of homelessness and housing instability is critical for efforts to resolve homelessness in this population, and future research should include their experiences.

### Conclusions

Our findings point to a critical need to move away from the predominant crisis-driven model found in many U.S. systems and toward the establishment of a comprehensive, cohesive health ecosystem in which all individuals are supported to live and thrive. A burgeoning literature is outlining components of distributed healthcare delivery ecosystems (Ritchie & Leff, 2022) and articulating central assumptions of ecological perspectives, including interdependence and multidirectional influences between individuals and communities (Boivin et al., 2022). The present study’s findings can be used to extend this work by investigating models of care that effectively reduce or eliminate system churn and foster community cohesion and a sense of belonging among and between all members of the community.
